# Complete Genome Sequence of Schaalia turicensis Strain CT001, Isolated from a Patient with Gonococcal Urethritis in Thailand

**DOI:** 10.1128/MRA.00836-21

**Published:** 2021-12-16

**Authors:** Natakorn Nokchan, Thidathip Wongsurawat, Piroon Jenjaroenpun, Perapon Nitayanon, Chanwit Tribuddharat

**Affiliations:** a Department of Microbiology, Faculty of Medicine Siriraj Hospital, Mahidol University, Bangkok, Thailand; b Department of Biomedical Informatics, University of Arkansas for Medical Sciences, Little Rock, Arkansas, USA; c Division of Bioinformatics and Data Management for Research, Department of Research and Development, Faculty of Medicine Siriraj Hospital, Mahidol University, Bangkok, Thailand; University of Rochester School of Medicine and Dentistry

## Abstract

Schaalia turicensis, a Gram-positive bacillus, is a potential pathogen in genital infections. Here, we report the complete genome sequence of S. turicensis strain CT001, which was coisolated with Neisseria gonorrhoeae. Comprehensive analysis revealed the presence of a composite transposon carrying an imperfect class 1 integron in S. turicensis.

## ANNOUNCEMENT

Schaalia turicensis is a Gram-positive, filamentous bacillus that is a commensal of the oral cavity, gut, skin, and female urogenital tract (1, 2), occasionally causing infections (3). S. turicensis was inadvertently recovered from a −80°C stock culture (30% glycerol in brain heart infusion broth) from a male patient with gonorrhea at Siriraj Hospital, Thailand, in 2010 after culturing on chocolate agar at 35°C ± 2°C in a 5% CO_2_ incubator for 48 h. During PCR screening of the class 1 integron-integrase gene in the Neisseria gonorrhoeae stock culture, only the pinpoint colonies showed positive results. These small colonies were confirmed to be S. turicensis by 16S rRNA gene sequencing using primers 27F (AGAGTTTGATCCTGGCTCAG) and 1392R (GGTTACCTTGTTACGACTT). The forward and reverse Sanger sequences were assembled (BioEdit v7.2.5), with sequence identity of ≥97% and ≥99% by BLASTn for the genus and species, respectively (4).

The S. turicensis genomic DNA (5-day culture) was extracted with the QIAamp PowerFecal Pro DNA kit (Qiagen) and sequenced using Oxford Nanopore Technologies (ONT) and Illumina platforms to obtain a complete genome sequence of the S. turicensis isolate. The quality control and *de novo* assembly steps for the S. turicensis isolate were modified from reference 5. For ONT sequencing, library preparation was done using the rapid barcoding sequencing kit (SQK-RBK004) without DNA size selection prior to sequencing with a flow cell (vR9.4/FLO-MIN106; ONT) using the MinION Mk1B sequencer (ONT) for 48 h. The raw signals from the sequencer were base called and demultiplexed using Guppy v3.2.4, followed by additional adapter trimming using Porechop v0.2.4 (https://github.com/rrwick/Porechop). The filtering of ONT raw reads based on a mean quality score of 8 was undertaken using NanoFilt v2.5.0 (6), and only reads with lengths of >1,000 bases were stored for the *de novo* assembly. For Illumina sequencing, 150-bp paired-end libraries were constructed with the NEBNext Ultra II DNA library preparation kit (New England Biolabs) and sequenced with a NovaSeq 6000 sequencer (Illumina). Reads were trimmed to exclude adapter sequences and filtered to obtain high-quality reads using fastp v0.19.5 (7). The read *N*_50_ was computed using the assembly-stats tool (https://github.com/sanger-pathogens/assembly-stats). Hybrid assembly of the ONT and Illumina reads was conducted using Unicycler v0.4.4 (8), generating a single circular chromosome of 1,912,310 bp (GC content of 57.1%). The error correction, circularization, and rotation (using the *dnaA* gene as the starting point) of the assembled genome were also performed using Unicycler v0.4.4. The quality of the draft genome was examined using QUAST v5.0.2 (9). The GC content was calculated using the GC Content Calculator (https://www.sciencebuddies.org/science-fair-projects/references/genomics-g-c-content-calculator). The default parameters were used for all software. The complete genome sequence was annotated by the NCBI Prokaryotic Genome Annotation Pipeline (PGAP) v4.11 (10).

The complete genome sequence of the S. turicensis isolate confirmed the species and the presence of a class 1 integron located within a 24.5-kb composite transposable element flanked by IS*6100*, registered as Tn*7083* via the Transposon Registry (11).

This nonclinical research study was permitted by the Siriraj Hospital institutional review board under certificates of approval Si479/2015 and Si720/2018.

### Data availability.

The complete genome sequence has been deposited in GenBank under BioProject accession number PRJNA607374. Accession numbers and genome features are summarized in Table 1.

**TABLE 1:** Genome characteristics and accession numbers for Schaalia turicensis strain CT001, isolated from a patient with gonococcal urethritis in Thailand

Parameter	Finding
BioSample accession no.	SAMN14131124
SRA accession no.	
Illumina	SRR11108583
ONT	SRR11108582
*N*_50_ (bp)	
Illumina	150
ONT	8,771
Total no. of reads	
Illumina	14,240,000
ONT	258,300
GenBank accession no.	CP048928
Genome size (bp)	1,912,310
Genome coverage (×)	921.7
GC content (%)	57.1
PGAP annotation	
Total no. of genes	1,690
Total no. of coding DNA sequences	1,630
